# Recent Progress of BODIPY Dyes With Aggregation-Induced Emission

**DOI:** 10.3389/fchem.2019.00712

**Published:** 2019-10-25

**Authors:** Zhipeng Liu, Zhiyong Jiang, Ming Yan, Xiaoqing Wang

**Affiliations:** ^1^College of Science, Nanjing Forestry University, Nanjing, China; ^2^College of Materials Science and Engineering, Nanjing Forestry University, Nanjing, China; ^3^Key Laboratory of Flexible Electronics (KLOFE) & Institute of Advanced Materials (IAM), Jiangsu National Synergetic Innovation Center for Advanced Materials (SICAM), Nanjing Tech University (NanjingTech), Nanjing, China

**Keywords:** BODIPY, aggregation-induced emission, fluorescence, bioimaging, sensor

## Abstract

With the development of organic optoelectronic materials and bioimaging technology, to exploit organic luminescent materials with high luminescent efficiency in aggregation-state has become a research hotspot. BODIPYs have become one of the research objects of this kind of material because of their obvious advantages. This review focuses on the design and synthesis of AIE-type BODIPYs, the mechanism of AIE properties and their applications in recent years. Through classification, analysis, and summary, this review aims to explore the structure-activity relationship of AIE-type BODIPYs and to provide ideas for the further design and potential applications of AIE-active fluorescent materials.

## Introduction

Organic luminescent materials (OLMs) are widely used in chemo/biosensors and light-emitting devices in light of their rich advantages, which include great diversity, easily modified structures, rich colors, and low environmental pollution (Chan et al., 2012; Uoyama et al., 2012; Yan et al., 2018). Since most of these applications heavily depend on their luminescent capabilities in the condensed state, the development of luminophores with excellent photophysical properties in the aggregation state is highly required. Traditional organic fluorescent dyes with a π-conjugated structure show excellent luminescent properties in dilute solution but become weakly or non-emissive in high concentration solutions or the aggregation-state, which is called the aggregation-caused emission quenching (ACQ) effect (Förster and Kasper, 1954). This effect is due to the collision between the ground state and the excited state of the fluorescent molecule at high concentration, which leads to the non-radiation deactivation process, or because the strong interaction between the planar π-conjugated structures leads the formation of excimers or exciplexes, and the energy of the excited state decays through the non-radiative form. The ACQ effect greatly limits the practical application of OLMs because the aggregated states are unavoidable for both light-emitting devices and fluorescent sensors.

**Figure 1:**
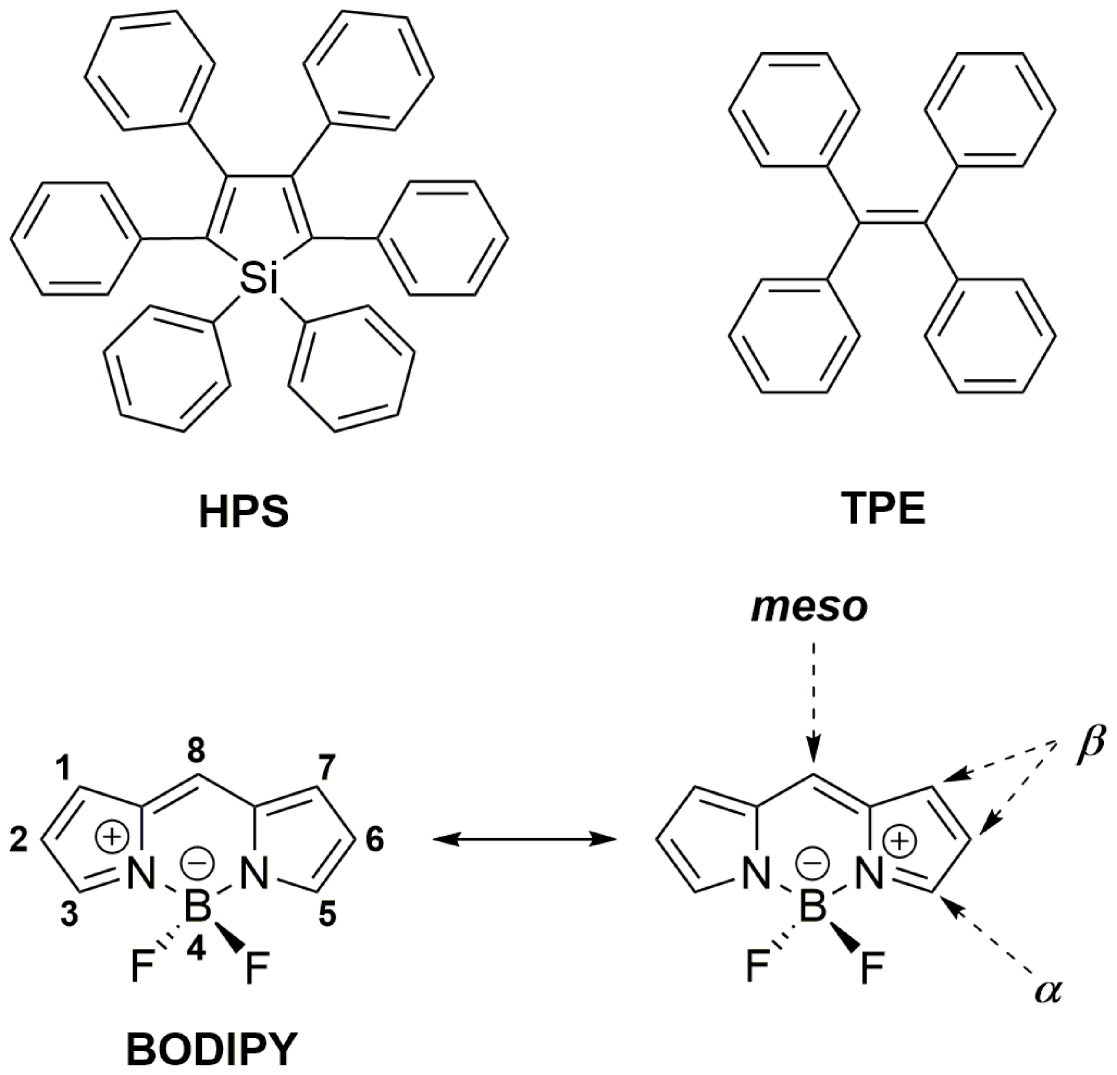
Chemical structures of HPS, TPE, and BODIPY.

Aggregation-induced emission (AIE), which was first introduced by Tang et al. has been widely accepted as a novel strategy to mitigate the ACQ effect on OLMs (Mei et al., 2015). Generally, AIE molecules such as 1,1,2,3,4,5-hexaphenylsilole (HPS, Figure 1) and tetraphenylethylene (TPE, Figure 1) usually possess highly twisted structures and show weak fluorescence in diluted solutions due to non-radiative transition induced by intramolecular motion (IM) in their excited state. In the aggregation state, such IM progress is effectively suppressed, resulting in their enhanced emission. Additionally, the highly twisted structure can effectively inhibit the π-π interactions between AIE molecules, which is conducive to improving their solid-state luminescence efficiency. Based on the widely accepted restriction of intramolecular motion (RIM) mechanism, AIE materials, including not only various newly designed molecules but also classical fluorophores including coumarins, pyrene, squaraines, cyanies, perleneimides, and BODIPYs, have been developed and applied in bioimaging, data encryption/decryption, OLEDs, and stimuli-responsive materials (Mei et al., 2015; Kokado and Sada, 2019).

As a classical luminophore, BODIPY dyes (boron dipyrromethene and its analogs, Figure 1) have achieved great development in the field of fluorescent sensing and bioimaging because of their excellent photophysical properties, including large molar extinction coefficients, high fluorescence quantum yields, tunable emission from visible light to near-infrared (NIR), and high photo- and chemical-stability (Loudet and Burgess, 2007; Boens et al., 2012; Lu et al., 2014; Kowada et al., 2015; Fan et al., 2016; Ge and O'shea, 2016; Wang et al., 2017). Unfortunately, in contrast to their excellent luminescence in a solution, most BODIPYs suffer from the ACQ effect and show weak fluorescence in the aggregation state. This is mainly because of the self-absorption and strong intermolecular interactions (π-π stacking, etc.) induced by their small Stokes shift and planar π-conjugated structures. These problems greatly restrict the further applications of BODIPYs as solid-state emitters. Therefore, the development of BODIPYs with aggregation-state fluorescence has received intense attention in the past decades.

AIE has been proved to be an efficient strategy for the construction of BODIPYs with efficient fluorescence in the aggregation state. A number of AIE-active BODIPYs have been rationally designed by various strategies, such as the direct integration of AIE molecules with the BODIPY skeleton (Hu et al., 2012; Gomez-Duran et al., 2015), J-type aggregation (Choi et al., 2014; Kim et al., 2015), and dipyrromethene bidentate ligand modification (Yang et al., 2012; Wang et al., 2015b). Taking the advantages of AIE, the intense aggregation-state fluorescence of BODIPYs has been successfully achieved. Moreover, their application as aggregation-state emitters for bioimaging, stimuli-response switches as well as OLEDs has been demonstrated (Mei et al., 2015; Baysec et al., 2018; Che et al., 2019). This mini review focuses on providing an overview of the design, mechanism and application of AIE-active BODIPYs and BODIPY analogs so as to facilitate their future application in the solid-state luminescence field. For the convenience of explanation, AIE-active BODIPYs are divided into two categories: one is based on the boron dipyrromethene platform (classical BODIPYs); the other is BODIPY analogs based on heterocycle-based bidentate chelates. The key photophysical data of each compound discussed are listed in Table 1. Moreover, boron difluoride complexes based on β–diketonate, ketoiminate, and diiminate will not be discussed in this review. A review that summarizes the photophysical properties and applications of these complexes would be helpful to readers (Tanaka and Chujo, 2015).

**Table 1:** Compilation of the photophysical data of BODIPY dyes with AIE.

	**In solution**				**In H**_****2****_**O-THF mixture**		**In solid-state**	
**Dye**	**solvent**	**λ_**abs**_ (nm)*^a^* (ε/M^**−1**^ cm^**−1**^)**	**λ_**em**_*^b^* (nm)**	**Φ_**f**_ (%)**	**λ_**em**_ (nm)*^b^* (*f*_**w**_)**	**Φ_**f**_ (%)**	**λ_**em**_*^b^* (nm)**	**Φ_**f**_ (%)**
1	THF	-	650	0.2	618 (90)	-	-	5
2	THF	-	640	0.3	640 (90)	-	-	27
3	THF	-	529	0.1	600 (90)	-	-	7.5
4	THF	536	567	59	569 (80)	53	-	-
5	THF	572 (54600)	630	2.9	630 (90)	3.9	642	10
6	THF	619 (26400)	697	6.4	697 (90)	2.32	706	6.9
7	THF	665 (80100)	690	42	690 (90)	-	690	1.3
8	THF	513	663	6.1	645 (99)	-	-	-
9	THF	498	586	0.74	700 (95)	-	-	-
10	THF	515	688	1.1	658 (99)	-	-	-
11	THF	501	754	0.27	-	-	-	-
12	THF	540	641	3	-	4	-	-
13	THF	513	633	4	-	10	-	-
14	THF	536	636	5	-	5	-	-
15a	CH_2_Cl_2_	550 (50000)	592	80	-	-	630 ± 4	-
15b	CH_2_Cl_2_	587 (48000)	618	81	-	-	636 ± 4	-
15c	CH_2_Cl_2_	589 (54000)	618	86	-	-	636 ± 4	-
15d	CH_2_Cl_2_	589 (47000)	619	87	-	-	636 ± 4	-
16	THF	514.5	525	-	-	-	537*^g^*	-
17a	CH_3_CN	549 (46310)	620	0.3	625 (99)*^d^*	6	-	-
17b	CH_3_CN	509 (87180)	535	0.3	587 (99)*^d^*	7	-	-
17c	CH_3_CN	509 (92160)	537	0.4	594 (99)*^d^*	-	-	-
17d	CH_3_CN	501 (85070)	536	0.8	557 (99)*^d^*	1.57	-	-
18a	EA*^c^*	503 (84000)	511.5	94	-	-	-	-
18b	EA*^c^*	503 (80000)	511	90	-	-	-	-
18c	EA*^c^*	503 (86000)	511	90	-	-	-	-
19a	EA*^c^*	503 (69000)	514.5	89	-	-	-	-
19b	EA*^c^*	503.5 (53000)	516.5	98	-	-	-	-
20a	CH_2_Cl_2_	507	514	2	575 (45)*^e^*	6.6	592	3
20b	CH_2_Cl_2_	502	515	2	-	-	595	5
21a	CH_2_Cl_2_	506	512	75	565 (50)*^e^*	32	609	16
21b	CH_2_Cl_2_	501	512	22	-	-	576	18
22a	CH_2_Cl_2_	508	515	40	545 (75)*^e^*	3.2	585	13
22b	CH_2_Cl_2_	502	515	13	-	-	590	28
23	CHCl_3_	484 (12882)	515	5	515 (96)	23	515*^g^*	14
24	THF	668 (128000)	683	0.8	683 (90)*^f^*	21.9	-	-
25	THF	677 (115000)	697	1.1	697 (90)*^f^*	18.8	-	-
26	THF	440	473	12	610 (90)	-	540*^g^*	-
27a	CH_2_Cl_2_	432	538	7	-	-	525*^g^*	20
27b	CH_2_Cl_2_	413	512	6	-	-	497*^g^*	52
27c	CH_2_Cl_2_	404	497	4	-	-	508*^g^*	19
27d	CH_2_Cl_2_	408	506	4	-	-	49 *^g^*	5
27e	CH_2_Cl_2_	384	545	3	-	-	515*^g^*	6
28a	THF	434 (47000)	514	<1	549 (90)	<1	573	2
28b	THF	398 (57000)	524	<1	564 (90)	1	543	1
29a	THF	305 (33000), 420 (76000)	506	<1	523 (90)	1	541	10
29b	THF	292 (35000), 394 (41000)	556	<1	562 (90)	<1	560	1
30a	hexane	380 (43700)	440	<1	495 (80)	-	495	26
30b	hexane	402 (25800)	499	41	-	-	503	60
31a	CH_2_Cl_2_	391 (33300)	429	2	-	-	525	13
31b	CH_2_Cl_2_	388 (24000)	426	1	-	-	488	15
31c	CH_2_Cl_2_	459 (70800)	529	78	-	-	629	20
32a	CH_2_Cl_2_	362 (16000)	450	<1	450 (90)	1	460	53
32b	CH_2_Cl_2_	387 (12000)	471	<1	475 (90)	1	494	46
33a	THF	373 (140000)	462	3	463 (90)	13	498	10
33b	THF	348 (370000)	441	1	443(90)	9	459	44
33c	THF	350 (310000)	437	2	440 (90)	23	463	38
33d	THF	367 (390000)	522	10	524 (90)	15	491	37
34a	CHCl_3_	459 (25119)	475	92	-	-	580	2
34b	CHCl_3_	452 (15848)	467	81	-	-	537	9.1
34c	CHCl_3_	510 (39810)	605	30	-	-	624	1.8
34d	CHCl_3_	466 (25119)	482	78	-	-	523	22
34e	CHCl_3_	525 (39810)	617	10	-	-	620	9.3
35a	THF	392 (19000)	447	1	480 (99)	7	494	29
35b	THF	397 (19300)	438	3	576 (99)	16	547	27
35c	THF	409 (37700)	485	1	521 (80)	7	506	21
35d	THF	408 (35500)	448	2	545 (90)	20	555	23
35e	CH_2_Cl_2_	395 (34200)	458	<1	-	-	473	0.60
35f	CH_2_Cl_2_	409 (43800)	522	<1	-	-	518	0.27
36a	CH_2_Cl_2_	407 (20000)	471	<1	471 (99)	14	481	12
36b	CH_2_Cl_2_	416 (21000)	464	<1	518 (90)	<1	534	20
36c	CH_2_Cl_2_	448 (11000)	522	<1	522 (90)	35	538	26
36d	CH_2_Cl_2_	461 (14000)	513	<1	583 (90)	<1	577	10

a *Longest absorption band*.

b *Longest emission band*.

c *EA, ethyl acetate*.

d *Measured in CH_3_CN/H_2_O mixture*.

e *Measured in methanol/H_2_O mixture*.

f *Measured in CH_2_Cl_2_/Hexane mixture*.

g *Measured in the film state*.

## Classical BODIPYs with AIE

### AIE-Active BODIPYs Based on TPE

**Figure 2:**
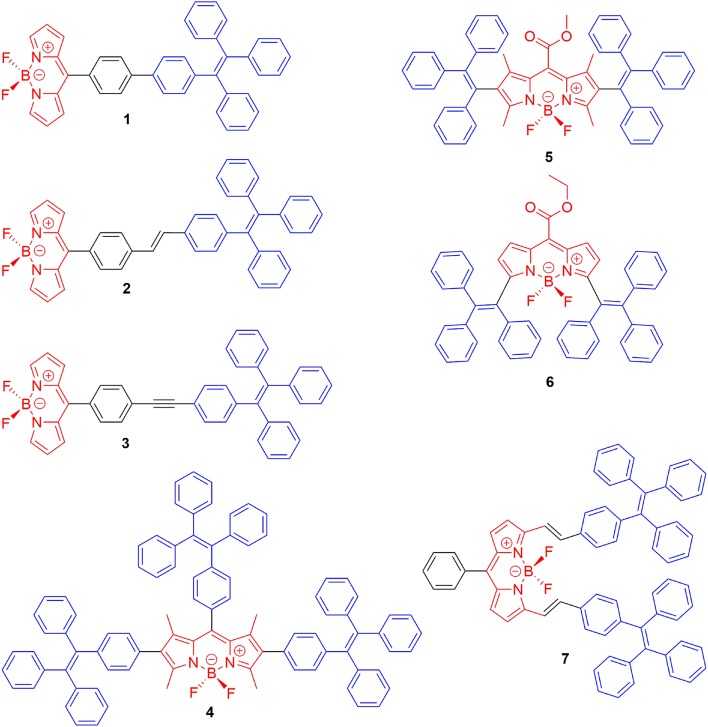
Chemical structures of compounds **1**–**7**.

Due to the planar π-conjugated structure of boron dipyrromethene core, strong intermolecular interactions such as π-π stacking and hydrogen bonds are usually observed in the aggregation state of BODIPYs, leading to distinct emission quenching. In order to suppress the strong intermolecular interactions, the well-known AIE luminescent element, TPE, has been successfully integrated with BODIPYs; thus, both AIE and intense aggregation-state emission were achieved. Tang et al. first reported TPE-containing BODIPYs (**1**–**3**, Figure 2) with AIE effect (Hu et al., 2012). In compounds **1**–**3**, TPE was simply introduced to the *meso*-position of the BODIPY core *via* a palladium-catalyzed cross-coupling reaction. In tetrahydrofuran (THF)-water mixture, both locally excited (LE) state, and twisted intramolecular charge transfer (TICT) state emission bands were observed in the emission spectra of compounds **1**–**3**. Compound **1** showed ACQ with the increment of the fraction of water (*f*_*w*_) in THF. In contrast, the intensity of the TICT emission band of compounds **2** and **3** was increased dramatically and accompanied by the decrement of the LE emission band. Meanwhile, the fluorescence quantum yields (*Φ*_f_) of compounds **2** and **3** in the solid state were determined to be 27 and 7.5%, respectively, which are higher than those of obtained in THF solution (*Φ*_f_ < 1%). Clearly, the relative stronger TICT effect of compounds **2** and **3** compared to compound **1** should be responsible for their different AIE and ACQ behavior.

The AIE behavior of TPE-BODIPY is highly dependent on the position and the number of TPE units attached to the BODIPY core. For example, Wu et al. (Chua et al., 2015), Scherf et al. (Baysec et al., 2018), and Atilgan et al. (Baglan et al., 2013) have reported that BODIPY derivatives with TPE or triphenylethene units at 2,6- or 2,6,8-positions (**4**–**5**, Figure 2) could effectively inhibit ACQ and act as aggregation-induced emission enhancement (AIEE). However, the conjugating of TPE at 3,5-position of the BODIPY core resulted in large π-conjugated structures with ACQ (**6**–**7**) (Gomez-Duran et al., 2015).

### AIE-Active BODIPYs Based on Triphenylamine (TPA)

**Figure 3:**
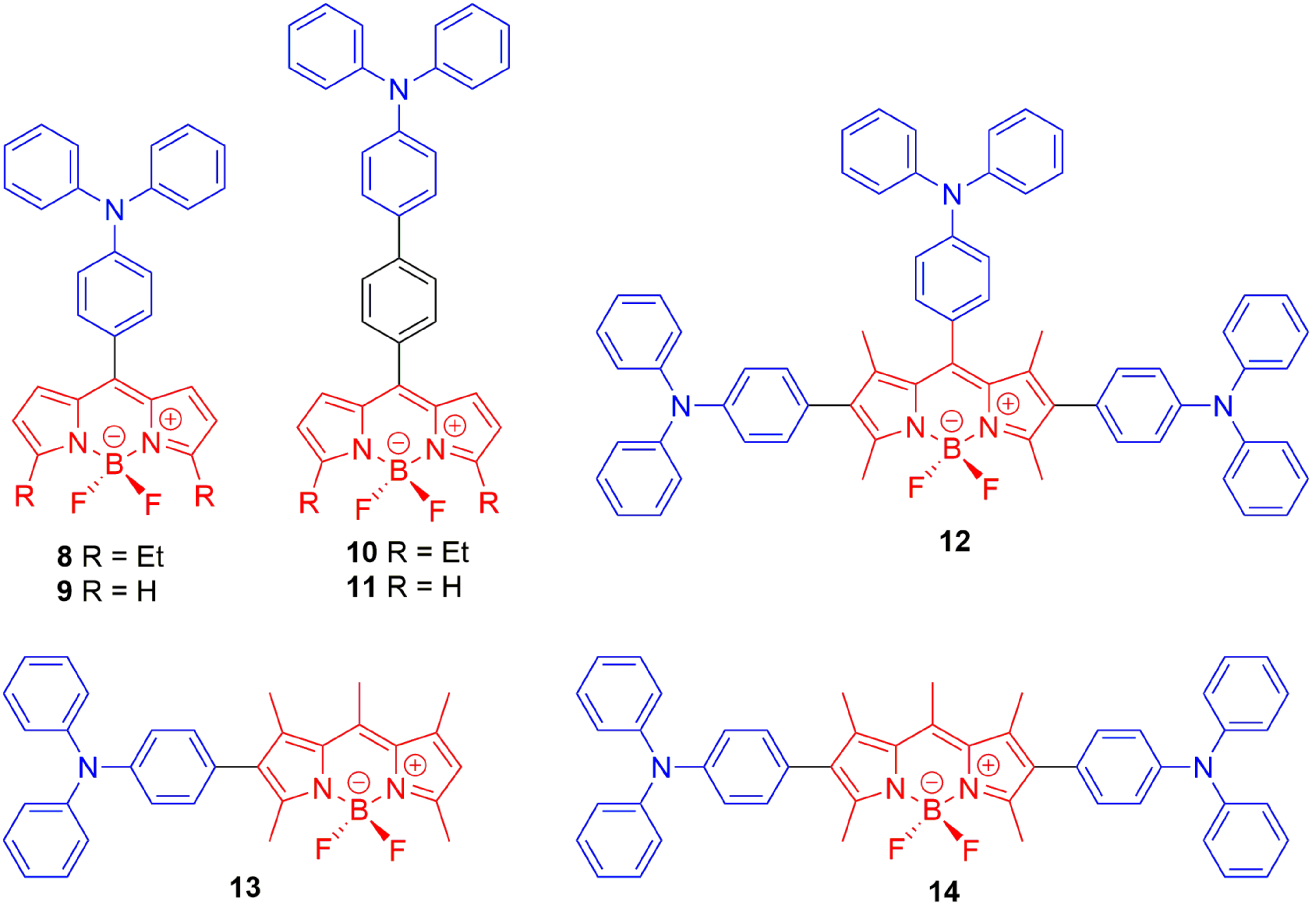
Chemical structures of compounds **8**–**14**.

**Figure 4:**
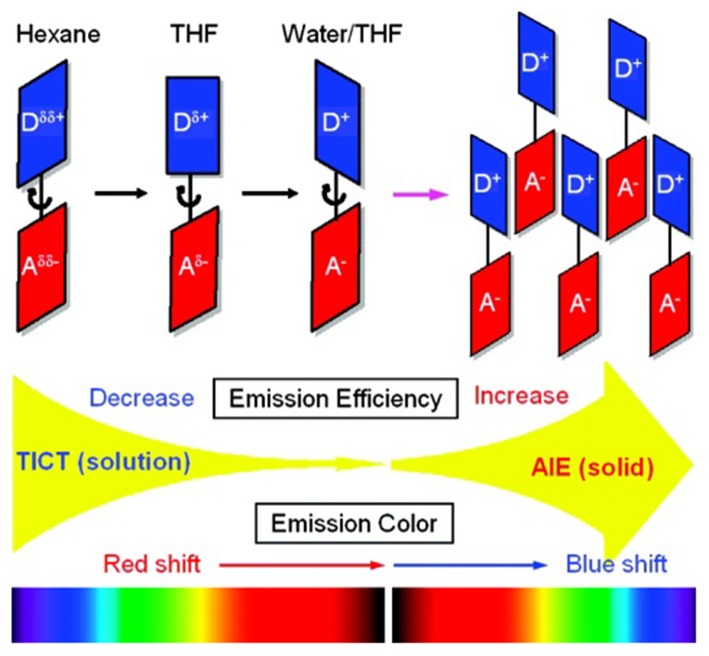
Proposed mechanism for TICT and AIE behaviors in AIE-active BODIPYs based on TPA (Reprinted from Hu et al., 2009. Copyright 2009 American Chemical Society).

Designing the propeller-shaped BODIPY molecules to consist of electron donor (D) and acceptor (A) units is another method to hinder the ACQ effect. Tang et al. developed a group of AIE-active BODIPYs with a D-A structure (**8**–**11**, Figure 3). Due to the strong electronic interaction between TPA (D) and BODIPY (A), compounds **8**–**11** displayed TICT and AIE properties. When the water was added to the THF solution of **8**–**11**, the LE emission intensity decreased with an increment of *f*_*w*_, accompanied by the red-shift of emission. This progress is mainly dominated by the polarity effect. However, when the *f*_*w*_ reached the point of aggregation, the rotation of the aromatic rings was efficiently restricted, resulting in blue-shifted emission, and AIE (Figure 4) (Hu et al., 2009; Lager et al., 2009). Moreover, compounds **12**–**14** with TPA unit incorporated into the 2-, 2,6-, 2,6,8-positions showed more enhanced TICT effect than compounds **8**–**11**, and only aggregation-induced emission enhancement of TICT was observed (Bui et al., 2019).

### AIE-Active BODIPYs Based on J-aggregation

Most BODIPYs tend to form H-type (face to face) aggregates in the aggregated state, which leads to fluorescence quenching. Recent studies indicated that the packing of BODIPYs could be engineered to favor the formation of J-type (head to tail) aggregates, and they would then give out red-shifted emission in comparison to their respective monomers in solution. Under this aggregation, the transition dipoles of the monomers aligned in a coplanar inclined way with a slip angle <54.7° to form dimers, trimers or even larger J-aggregates (Wurthner et al., 2011; Choi et al., 2014; Tian et al., 2018).

**Figure 5:**
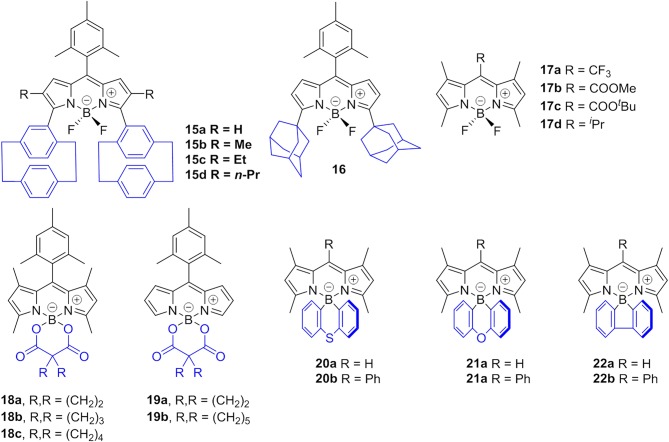
Chemical structures of compounds **15**–**22**.

**Figure 6:**
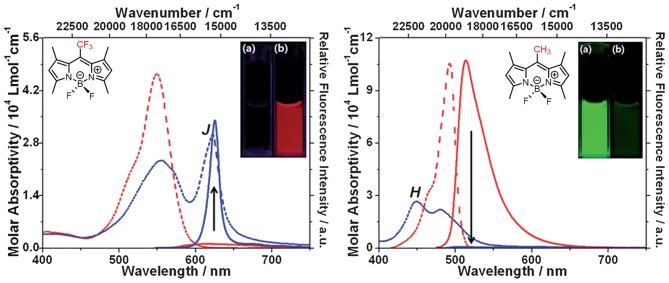
Different aggregation behavior of **17a** (2 × 10^−5^ mol L^−1^) and it's analog with a methyl group at the *meso*-position in acetonitrile and acetonitrile-water mixtures (1:99, v: v). Dotted and solid lines refer to the absorption and emission spectra of BODIPYs, respectively (adapted with permission from Choi et al., 2014, Copyright 2014, Royal Chemistry Society).

Johansson et al. first evidenced the formation of non-fluorescent BODIPY H-dimers in double-labeled proteins and emissive J-dimers in labeled lipid vesicles (Bergström et al., 2002). Vu et al. found that bulky substituents at the 3- and 5-positions of the BODIPY core, such as paracyclophane (**15**, Figure 5) (Vu et al., 2009) and the adamantyl group (**16**) (Vu et al., 2013), could facilitate the formation of emissive J-aggregates in the aggregation state. To elucidate the factors that govern the formation of emissive J-aggregates of BODIPYs, Kim et al. carried out a systematic study of the substitution effect on the *meso*-position (Choi et al., 2014; Kim et al., 2015). For 1,3,5,7-tetramethyl derivatives, the *meso*-substituents **17**, that are -CF_3_, -COOMe, -COO^*t*^Bu, and -^*i*^Pr, demonstrably formed emissive J-aggregates. Meanwhile, other *meso*-substituents, such as -CH_3_, -CHO, -CN, and -Cl, exhibited the ACQ effect or were fluorescent in the solid state without forming J-aggregates (Figure 6). The formation of emissive J-aggregates is quite sensitive to minute structural changes. J-aggregations were not encountered in the closely related 3,5-dimethyl derivatives. Both the electron-withdrawing *meso-*substituents and flanking methyl groups are necessary for the formation of emissive BODIPY J-aggregates. Moreover, by using the AIEE-type *meso*-ester-substituted BODIPY probe **17b**, they realized the need to detect specifically HOBr generated by eosinophil peroxidase (EPO) for a clean turn-on signal: the red emissive (621 nm) J-aggregates of 2,6-dibrominated **17b** self-assembled into orange emissive (581 nm) J-aggregates (Kim et al., 2018).

Besides the abovementioned J-aggregation tuning tuned *via* variation the *meso*-substitutions, the modification of BF_2_ moiety with a diacyloxyl or diaryl substituent should be another potential strategy. For example, AIE behavior induced by J-aggregation of BODIPY in pure organic solvents was described by Chiara et al. in *O*-BODIPYs with a B-spiranic 4,4-diacyloxyl substitution pattern (**18**, **19**, Figure 5). The high conformational rigidity of this design along with the orthogonal disposition of the B-diacyloxyl substituent and the *meso*-aryl group were analyzed to be the key factors of the J-aggregation process (Manzano et al., 2016). Wang et al. investigated spiro-BODIPYs with a diaryl chelate unit that could form J-aggregates in the alcohol-water mixture. The J-aggregates of **20a** showed increased emission efficiency while those of **21a** and **22a** indicated decreased emission efficiency, suggesting that the change in emission intensity is not a reliable indicator for the formation of J-aggregates. An important detail to mention is that similar structures substituted by phenyl at *meso-*position (**20b**–**22b**) were not observed in the J-aggregation formation of the alcohol-water mixture or in the tetrahydrofuran-water mixture (Yuan et al., 2017).

## Bodipy Analogs with AIE

AIE strategy, relying on the RIM, has produced numerous systems with high emission in the aggregation state. Except for classical BODIPYs, the development of new members of BODIPYs family viz. BODIPY analogs with AIE-active will undoubtedly contribute to a better understanding of the phenomena and lead to novel applications.

**Figure 7:**
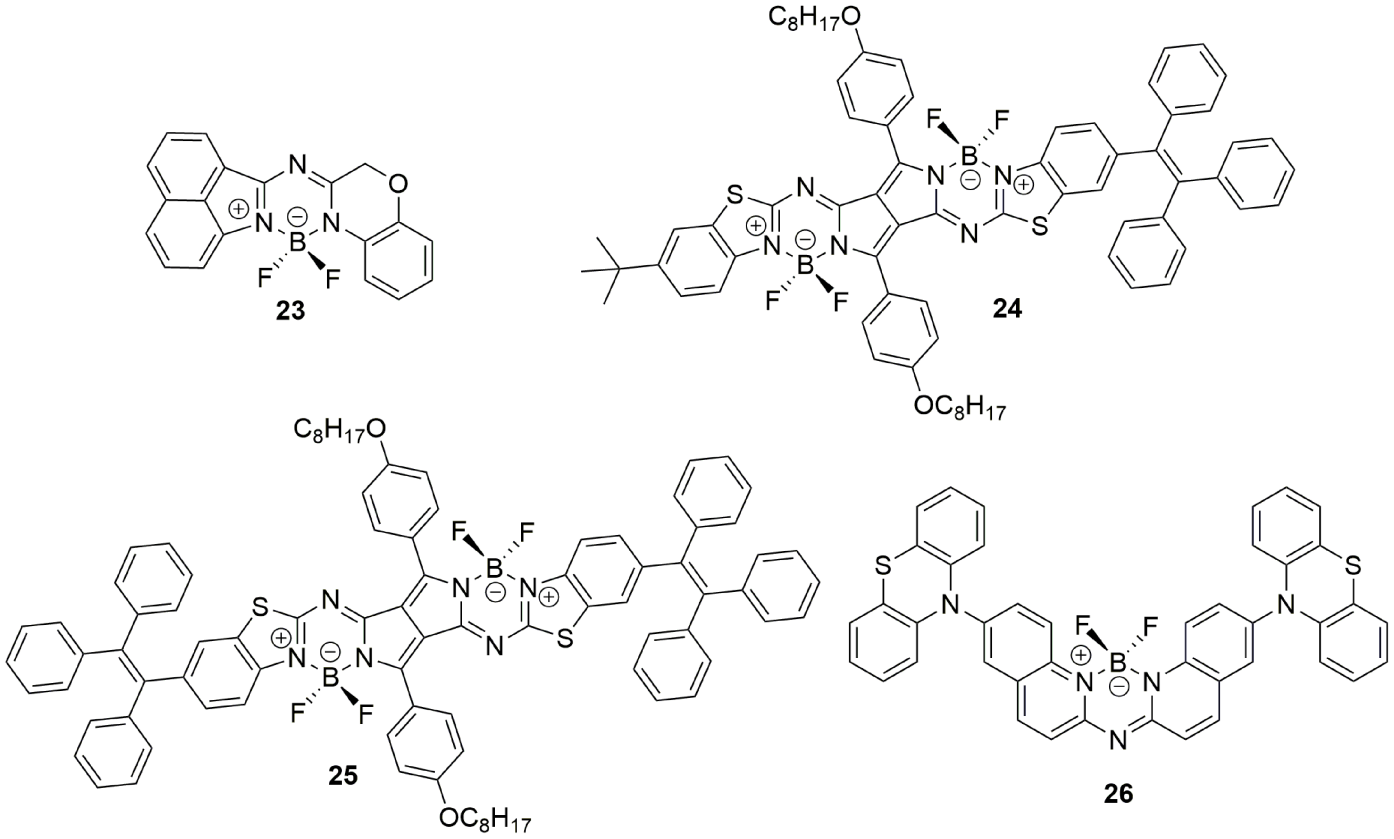
Chemical structures of compounds **23**–**26**.

In the process of elucidating the optical properties of benzo[*c, d*]indole-containing aza-BODIPYs, Kobayashi et al. found that the photophysical property of aza-BODIPYs could be tuned by incorporating heteroaromatic moieties in place of pyrrole or isoindole rings. Moreover, they reported the first aza-BODIPY (**23**, Figure 7) exhibiting AIEE behavior. Compound **23** showed weak fluorescence in a diluted solution (*Φ*_f_ = 2% in THF solution), however, fluorescence enhancement was observed both in film-state (drop-cast film, *Φ*_f_ = 14%) and aggregation-state (*f*_*w*_ = 90%, *Φ*_f_ = 23%). Clearly, the restricted molecular dynamics induced by the non-conjugated moiety should be responsible for such AIEE phenomenon (Shimizu et al., 2015).

Similar to the strategy mentioned in section AIE-active BODIPYs Based on TPE, the AIE property of NIR-emissive aza-BODIPYs based on a diketopyrrolopyrrole-benzo[*d*]thiazole ligand was realized by linking one or two TPE moieties to its planar π-conjugated structure (Li et al., 2017). Compounds **24** and **25** showed weak fluorescence with *Φ*_f_ of 0.7 and 0.4% in diluted dichloromethane solution. After the adding of hexane as a poor solvent to the dichloromethane solution, great fluorescence enhancement of around 690 nm was observed (21.9% for compound **24**, and 18.8% for compound **25**) due to the formation of high emissive aggregates. Moreover, the imaging ability of **25**-NPs, which was prepared from compound **25** and Pluronics 127, has been proved in HeLa cells.

By incorporating two phenothiazine units into the biquinoline-based ligand, an AIE-active aza-BODIPY with highly twisted structure (**26**, Figure 7) was reported by Zhu et al. (2014). Compound **26** showed weak blue emission at 480 nm in THF solution. The addition of water to the THF solution (*f*_*w*_ ≤ 50%) first induced emission quenching due to the enhanced TICT effect. Then, great red-shifted emission from 480 to 610 nm accompanied by emission enhancement was observed because of the formation of aggregates.

Besides the strategies of incorporation of AIE units into the BODIPY core and J-aggregation engineering, modification of the dipyrromethene bidentate to give BODIPY analogs with desymmetrized and propeller-shaped structure has also proved to be an efficient method to achieve AIE-active BODIPYs with high aggregation-state *Φ*_f_. Generally, these BODIPY analogs usually show a larger Stokes shift than classical BODIPY, which is helpful for suppressing the self-absorption in the condensed phase. Moreover, as a benefit of their high twisted structure, the strong π-π interaction can be efficiently avoided. Based on the above conception, various AIE-active BODIPY analogs with the propeller-shaped structure have been developed by replacing the dipyrrole units to various heterocycles such as pyridine, benzo[*d*]thiazole, quinoline, *etc*.

**Figure 8:**
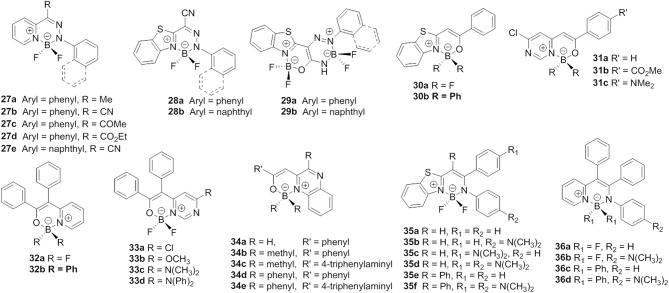
Chemical structures of compounds **27**–**36**.

Heterocycle-hydrazone-based boron difluoride complexes, which were first reported by Aprahamian et al. are a new class of AIE-active BODIPY analogs (**27**, Figure 8) (Yang et al., 2012). Due to the desymmetrized and propeller-shaped structure, compound **27a** showed weak fluorescence at 512 nm (*Φ*_f_ < 10%) with a large Stokes shift (101,010 cm^−1^) in dichloromethane. After restricting the intramolecular rotations, enhanced emission both in the film and crystalline state was observed. Most importantly, the AIE mechanism of pyridine-hydrazone-based boron difluoride complexes was rationalized by TD-DFT calculations (Qian et al., 2017). The calculated results demonstrated that the emission of these compounds was not generated from the S_1_ state but from the other excited states with higher energy (>S_1_). The authors also suggested that suppression of Kasha's rule should be the real mechanism responsible for emission in the solid state.

Inspired by the AIE-active BOIDPY analogs based on pyridine-hydrazone ligands, a group of new AIE-active monoboron (**28**) and bisboron (**29**) difluoride complexes was developed by adopting benzo[*d*]thiazole-hydrozone as the chelates (Duan et al., 2018). Similar to compound **27a**, these complexes showed weak emission (*Φ*_f_ < 1%) and large Stokes shifts (up to 7,400 cm^−1^) in a diluted solution and AIEE in the aggregation state. The AIE behavior of these complexes was investigated and found to be closely related to the RIR of the aromatic rings.

In consideration of the attractive properties of heterocycle-amidine and heterocycle-hydrazone ligands based BODIPY analogs, it is worth to further expand the family of these BODIPY analogs so as to develop more AIE-active functional materials. In this case, various BODIPY analogs based on heterocycle-enolate ligands have been successfully developed. Matsui et al. synthesized a boron difluoride complexes derivate from βbenzo[*d*]thiazole-enolate ligands (**30**, Figure 8) (Kubota et al., 2012). The desymmetrized structure and AIEE effect make these complexes show high emission with *Φ*_f_ up to 60% in the solid state. Based on this pioneering work, Matsui et al. also investigated the photophysical and AIE properties of monoboron and bisboron complexes based on pyrimidine-enolate ligands (**31**, Figure 8) (Kubota et al., 2013). By tuning the CT and conjugation effect, an intense solid-state emission maximum from 488 to 641 nm was achieved (*Φ*_f_ = 7–20%). Next, a number of propeller-shaped BODIPY analogs based on pyridine- (Wu et al., 2015), quinoxaline- (Liao et al., 2015), pyrimidine- (Qi et al., 2016), and benzo[*d*]thiazole-enolate (Gong et al., 2015) ligands (**32–34**, Figure 8) were reported by different groups. Like HPS and TPE, the propeller-shaped structure quenched the emission of these complexes in a diluted solution, and, after restricting the intramolecular rotations by aggregation, distinct AIE behavior was observed.

By reacting pyridine- and benzo[*d*]thiazole-enolate ligands with arylamine, our group developed a series of pyridyl- and benzothiazole-enamide *N*^∧^*N*-bidentate ligands, which facilitated the generation of a new family of propeller-shaped BODIPY analogs (Liu et al., 2015; Wang et al., 2015a,b). Similarly to the abovementioned compounds **27**–**34**, compounds **35** and **36** showed very weak emission in low-viscosity solvents and displayed AIE in the aggregation state. All of these compounds showed large Stokes shifts and very weak intermolecular interactions in the aggregation state, resulting in high *Φ*_f_. Moreover, the applications of these compounds as solid-emitters for acid gas and pressure sensing were also demonstrated.

## Applications of AIE-active BODIPYs

Although BODIPYs have gained great success in biological sensing and imaging, their application as the emitter in the aggregation state was rarely reported in the past decades. This obstacle would be well-solved by the rational designing of aggregation-state emissive BODIPYs and their analogs. Indeed, taking the advantages of AIE, the application scope of BODIPYs has been successfully expanded from solution state to aggregation state in recent years.

### Fluorescent Imaging

Fluorescence imaging technology has been demonstrated as a powerful tool for investigating biological processes in living cells and clinical diagnostics because of its high specificity and sensitivity, high resolution, and its nondestructive properties (Johnson and Spence, 2010). Numerous fluorescent materials such as fluorescent proteins, quantum dots, polymer dots, and small organic fluorescent molecules with photo-active or photoswitch properties have been developed (Lavis and Raines, 2008; Chan et al., 2012; Li et al., 2014). Compared with the above fluorescent materials, AIE materials have distinct advantages, including high emission at high concentration or in the aggregation state, low toxicity and good anti-photobleaching ability, which make them hold great potential as candidates for fluorescent imaging (Chen et al., 2016). As an important member of AIE materials, AIE-active BODIPYs have been employed as imaging agents in biological sensing and imaging.

Tang et al. first used the aggregates of compound **3** for fluorescence imaging in living HeLa cells. After staining living cells with the aggregates, both the LE emission, and TICT emission were detected (Hu et al., 2012). After that, the biocompatible AIE dots that were prepared from AIE-active BODIPY molecules were applied for intracellular fluorescent imaging. For example, red emissive nanoparticles of compound **4** were obtained by encapsulating compound **4** with 1,2-sistearoyl-sn -glycero-3-phosphoethanolamine-N-[methoxy(polyethylene glycol)-2000] (DSPE-PEG_2000_). Benefitting from their good biocompatibility and two-photon absorption and excited fluorescence (TPEF), these AIE NPs were further applied in TPEF cellular imaging and mouse brain blood vascular visualization, suggesting their potential application in TPEF sensing and imaging (Zhao et al., 2014).

**Figure 9:**
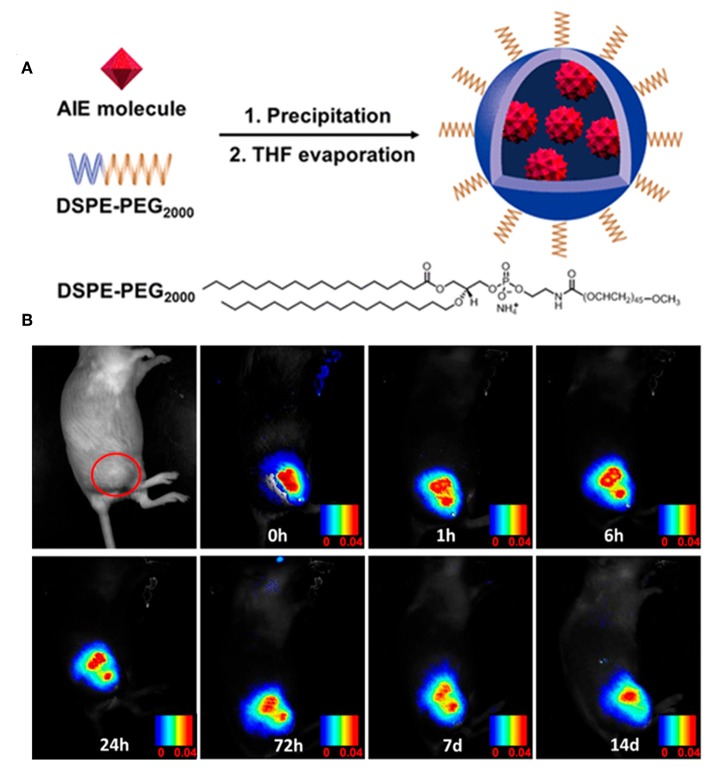
**(A)** Preparation of the AIE NPs of compound **12**. **(B)** *In vivo* confocal images of the tumor-bearing mouse with AIE NPs of compound **12** from day 0 to day 14 (adapted with permission from Che et al., 2019, Copyright 2019, Royal Chemistry Society).

Very recently, the efficient imaging capabilities of TPA- and carbazoyl-based AIE-active BODIPYs have also been reported by Tang and Su. After fabricating in the presence of DSPE-PEG_2000_, the NPs of compound **12** were obtained with intense far-red emission (around 650 nm) and excellent photostability. Moreover, these NPs showed an ultrafast cell staining time of a few seconds and excellent cell imaging ability. More importantly, these NPs can be used for long term imaging both *in vitro* and *in vivo* (Figure 9), demonstrating their great potential imaging abilities in the practical biological applications (Che et al., 2019).

### Mechanofluorochromic (MFC) Materials

**Figure 10:**
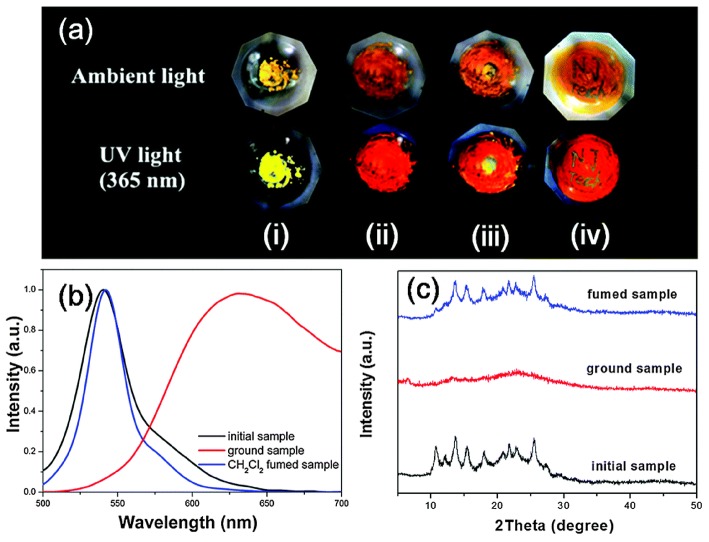
**(a)** Photographs of the powder of compound **26** before and after grinding under ambient and light UV light (365 nm): (i) crystalline powder, (ii) ground powder, (iii) ground powder upon adding of a drop of CH_2_Cl_2_, and (iv) the letters “NJ Tech” were written on the ground powder using CH_2_Cl_2_. **(b)** Emission spectra and **(C)** PXRD patterns of the powder, after being ground and CH_2_Cl_2_-fumed (Reprinted from Zhu et al., 2014, Copyright 2014, Royal Chemistry Society).

MFC materials that change their luminescence upon mechanical grinding/shearing have been attracting a great deal of interest owing to their promising applications (Sagara et al., 2016). Generally, AIE molecules with a strong twisted skeleton with rotatable aryl units, resulting in the stacking of loose molecules in the crystal state, can be easily destroyed by mechanical stimulation, resulting in a change of luminescence color. Thus, twisted π-conjugated AIE-active BODIPY analogs have been used as potential candidates for promising MFC materials. For example, the yellow powder of compound **26** showed bright fluorescence with λ_em_ at 540 nm. After grinding with the motor, the yellow emissive powder immediately changed its emission color to red (λ_em_ = 635 nm), resulting in a 95 nm red-shift of emission. Moreover, the mechanic-induced color change can be switched back via dichloromethane fuming. The crystalline-amorphous-crystalline state transformation of **26** during the grinding and fuming stimuli processes has been demonstrated by powder X-ray diffraction (PXRD) (Figure 10) (Zhu et al., 2014).

**Figure 11:**
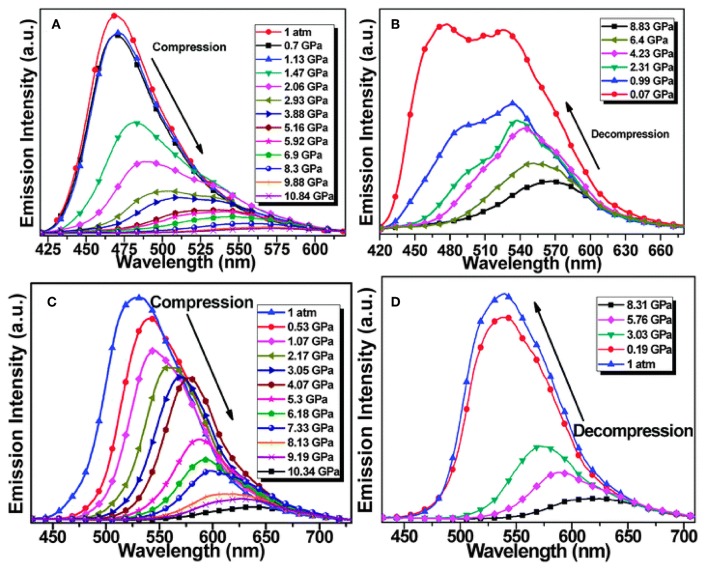
Emission spectra of compounds **35e (A,B)** and **35f (C,D)** upon the compressing **(A,C)** and following decompressing processes **(B,D)** (Reprinted from Wang et al. (2015a), Copyright 2015, Royal Chemistry Society).

Upon mechanical grinding or hydrostatic compression, compounds **35e** and **35f** displayed red-shift emission under high pressure, while **35f** with ICT effects showed a more sensitive piezochromic response at low pressure (<1.5 GPa), which implied that the pressure-dependent π-π intermolecular interaction and the intramolecular CT effect were efficient in inducing piezochromic luminescence (Figure 11) (Wang et al., 2015a). The distinct piezochromic effect of **35f** at low compression pressure suggested that the propeller-shaped AIE luminophore with the ICT effect could be a valuable basis upon which to design MFCs with high sensitivity. In the process of studying pyrimidine-based BF_2_ complexes, **33a**–**d**, we found that only **33d** showed distinct luminescence change upon mechanical stimuli. **33d** underwent red-shift from 491 to 509 nm on mechanical grinding, while it recovered to the original state when exposed to dichloromethane vapor for 10 min. In addition to the XRD characteristic, we rationalized that the mechanochromism is attributed to the desymmetric propeller-shaped configuration and donor-acceptor character of **33d** (Qi et al., 2016).

### Gas Sensors

**Figure 12:**
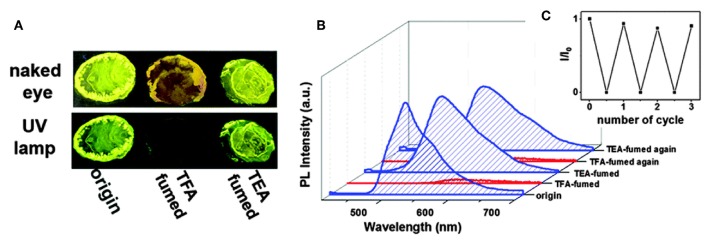
**(A)** Photographs of compound **34d** on sliding glass taken under ambient and UV light (λ_ex_ = 365 nm). **(B)** Emission spectra of compound **34d** after treating with TEA–TFA vapors in solid-state. **(C)** Recycling of the emission switching of the power of compound **34d** upon fuming with TFA and TEA vapors (Reprinted from Liao et al., 2015, Copyright 2015, Royal Chemistry Society).

Due to their strong fluorescent properties in the solid state, AIE-active BODIPYs and their analogs are proposed to be an ideal candidate for gas sensing. A number of compounds have been reported as the fluorescent switch for organic solvent, acid and base gases. For example, compounds **34a**–**e** possessed unusual acidochromic behavior triggered by acid vapor (Liao et al., 2015). After exposure to trifluoroacetic acid (TFA) vapors, the colors of **34a**-**e** turned obscure, and absorption spectra were red-shifted and accompanied by strong quenching of luminescence. The effect of fluorescence quenching upon acid fuming should be attributed to the synergistic effects of the protonation of nitrogen resulting from the pyrazine segment-induced push-pull effect and the changes of intermolecular packing and molecular conformation upon acid protonation (Figure 12).

Another example is that the intense solid-state emission of compound **26** could be switched by multiple external stimuli, including grinding, organic solvent vapors as well as acid and base vapors (Zhu et al., 2014). Fumigation of **26** with HCl–TEA vapors exhibited an off/on switching fluorescence effect. Based on the protonation–deprotonation stimuli luminescence property of **26**, a simple, convenient and efficient piece of technology for data encryption and decryption was designed. All these comprehensive investigations suggested that complex **26** was a very promising candidate for application in sensing, detection, and security protection.

The introduction of the *N,N*-dimethylamino group as an acid-sensitive group to the π-conjugated structure of AIE-active BODIPYs has been demonstrated to be an efficient strategy for achieving highly sensitive acidic vapor sensing. Taking compound **35b** as an example, when exposed to HCl vapors for a few seconds, **35b** exhibited blue-shifted emission with the color changing from yellow to cyan (547–518 nm). The protonated powder samples gradually recovered their original color and fluorescence when they were treated with NH_3_ vapor for a few minutes. Such an acidic/basic gas-triggered solid-state emission change was also observed in compounds **35d**, **36b**, and **36d** (Liu et al., 2015; Wang et al., 2015b).

## Summary and outlook

Among many organic fluorescent molecules, the synthesis of BODIPY fluorescent molecules and their analogs is relatively simple, and the advantages of photophysical properties are prominent, such as a high molar extinction coefficient, high quantum yield, tunable emission wavelength, and high stability. The methods of achieving AIE activity of classical BODIPY and BODIPY analogs mainly include linking AIE-active molecules on the chromophore core, the J-aggregation method, and designing fluorescent molecules into propeller-shaped structures. Because of their high luminescence efficiency in aggregates and solid-state, these molecules have been successfully applied to bioimaging, solid-state stimulus-responsive materials, OLEDs and other fields. However, some challenges still exist for the design and application of the AIE-active BODIPYs.

Fundamental understanding of the aggregation effect on photophysical property is not yet satisfactory. Mechanisms based on RIM and J-aggregation are generally applied in molecular design, and in some cases they do not work as well as expected yet. For example, integrating the TPE unit to BODIPY is generally thought to induce AIE, but ACQ behavior toward some BODIPYs bearing TPE units was reported (Gomez-Duran et al., 2015). On the other hand, J-aggregation of BODIPYs should result in emissive J-aggregates in the aggregation state; however, we recently demonstrated that the J-aggregation could generate multiple emissions across the red to NIR region (Tian et al., 2018).

Improving the fluorescent efficiency in the aggregation state is highly required. Although the ACQ effect of BODIPYs was suppressed by introducing an AIE unit, the aggregation-state *Φ*_f_ for most of AIE-active BODIPYs remained low, which restricted their further applications. Exploring a new strategy or a proper platform to achieve intense aggregation-state emission of BODIPYs remains a challenge.

There is huge scope in exploring AIE-active BODIPYs with NIR emission (700–1,700 nm). Compared to the large number of AIE-active BODIPYs with a short emission wavelength, successful examples of NIR emission are rather limited. Exploring the suitable building block and fine-tuning of the π-conjugated structures should be helpful for achieving NIR aggregation-state emission. Aggregation-state emissive BODIPYs, especially those with NIR-II (1,000–1,700 nm) emission, could have a bright future for *in vivo* and clinical imaging (Zhu et al., 2019).

Taken together, under the guidance of AIE, efficient aggregation-state emissive BODIPYs with diverse chemical structures and intriguing photophysical properties will be developed. These BODIPY derivatives will undoubtedly show their capabilities in various application fields.

## Author Contributions

ZL and XW designed this proposal, revised the manuscript, and determined the contents. ZJ and MY drew the chemical structures and prepared the figures. All authors contributed to the writing of the manuscript.

### Conflict of Interest

The authors declare that the research was conducted in the absence of any commercial or financial relationships that could be construed as a potential conflict of interest.
